# Supplementary material for: Regulation of the endosomal SNX27-retromer by OTULIN
Source: Nat Commun. 2019 Sep 20;10:4320. doi: 10.1038/s41467-019-12309-z (PMC6754446; doi:10.1038/s41467-019-12309-z)
Supplement: Supplementary file 1 — Supplementary Information [file 41467_2019_12309_MOESM1_ESM.pdf]

Supplementary Information

Regulation of the endosomal SNX27-retromer by OTULIN

Weber at al.

	Syringe	Cell	K_D (μM)	ΔH (kcal/mol)	TΔS (kcal/mol)	ΔG (kcal/mol)	N (stoichiometry)
SNX27 OTULIN	$SNX27_{PDZ}$	OTULINcat	0.031 ±0.014	-21.7 ±1.4	11.3 ±1.3	-10.4 ±0.3	0.91 ±0.16
	OTULINcat	$SNX27_{PDZ}$	0.014	-21.2	10.5	-10.7	1.02
	$SNX27_{PDZ}$	OTULINcat ΔETSL	n.b.d*				
	Ac-VRVCEETSL	$SNX27_{PDZ}$	1.10 ±0.035	-15.5 ±1.7	7.3 ±1.7	-8.1 ±0.01	1.07 ±0.18
SNX27 mutations	$SNX27_{PDZ}$ G64E	OTULINcat	0.34 ± 0.007	-15.2 ±0.4	6.4 ±0.4	-8.8 ±0.01	0.75 ±0.02
	$SNX27_{PDZ}$ G65A	OTULINcat	0.093 ± 0.019	-18.3 ±0.2	-8.7 ±0.1	-9.6 ±0.1	0.76 ±0.2
	$SNX27_{PDZ}$ R100E	OTULINcat	0.26 ± 0.15	-19.6 ±0.9	10.6 ±0.5	-9.1 ±0.4	0.89 ±0.21
	$SNX27_{PDZ}$ G64E, R100E	OTULINcat	1.09 ± 0.007	-14.0 ±0.1	5.9 ±0.2	-8.1 ±0.1	0.84 ±0.01
OTULIN mutations	$SNX27_{PDZ}$	OTULINcat E85R	0.84 ± 0.11	-18.0 ±0.6	9.6 ±0.5	-8.3 ±0.08	1.02 ±0.05
	$SNX27_{PDZ}$	OTULINcat D87R	0.31 ± 0.08	-19.8 ±1.1	10.9 ±0.9	-8.9 ±0.2	0.99 ±0.09
	$SNX27_{PDZ}$	OTULINcat E85R, D87R	2.07 ± 0.12	-17.2 ±0.8	9.4 ±0.8	-7.8 ±0.04	1.53 ±0.07
	$SNX27_{PDZ}$	OTULINcat D90R	0.12 ± 0.03	-20.9 ±0.6	11.4 ±0.6	-9.4 ±0.1	0.75 ±0.12
	$SNX27_{PDZ}$	OTULINcat E209R	0.05 ± 0.01	-24.0 ±4.2	14.0 ±4.0	-10.0 ±0.2	0.74 ±0.04
Other PDZs	[1]$SHANK1_{PDZ}$	OTULINcat	22.6	-9.3	3.3	-6.0	1.00
	[1]$SHANK2_{PDZ}$	OTULINcat	23.9	-10.2	3.8	-6.5	0.99
	[1]$SHANK3_{PDZ}$	OTULINcat	26.4	-8.6	2.3	-6.4	1.00
	$RhoGEF_{PDZ}$	OTULINcat	38.3	-5.5	-0.5	-6.0	0.96
	$PSD95_{PDZ2}$	OTULINcat	98.9	-3.8	-1.6	-5.5	0.91
	$NHERF1_{PDZ2}$	OTULINcat	35.6	-23.0	17.0	-6.1	1.14
	$PSD95_{PDZ3}$	OTULINcat	n.b.d*				
	$MUPP1_{PDZ}$	OTULINcat	n.b.d*				
	$Syntenin1_{PDZ1}$	OTULINcat	1710**	50.9	-54.7	-3.8	0.38
	$LNX2_{PDZ3}$	OTULINcat	57.1	-5.1	-0.7	-5.8	0.87
SNX27 VPS26A	VPS26A	$SNX27_{PDZ}$	27.5 ±7.6	-12.8 ±0.2	6.5 ±0.4	-6.1 ±0.1	0.81 ±0.19

Supplementary Table 1: Summary of ITC data

OTULIN_80-B_Fwd	AAGTTCTGTTTCAGGGCCCGTTAAGCGTAGCTCCTGAAATGGATATC
OTULIN_352-B_Rev	ATGGTCTAGAAAGCTTTAttaTAGACTGGTCTCCTCACACACTC
OTULIN_pHAGE_Fwd	TTCGATTCTAGAAGTCGGGGGACTATGCCCCAGC
OTULIN_pHAGE_Rev	CGATGTCGACTTATAGACTGGTCTCCTCACACACT
OTULIN_pEF_Fwd	CGATGGATCCAGTCGGGGGACTATG
OTULIN_pEF_Rev	CTAGCGGCCGCTTATAGACTGGTCTCCTC
VPS26a_1-Ncoi_Fwd	GCCCGAGAccatgggtATGAGTTTTCTTGGAGGCTTTTTTGGT
VPS26a_327-His6_KpnI_Rev	GCCCGAGAggtaccttattagtgatggtgatgatggtgCATTTCAGGCTGTTCGGCAGATG
VPS26A_pEF_Fwd	CGATGGATCCAGTTTTCTTGGAGG
VPS26A_pEF_Rev	CGATGCGGCCGCTTACATTTCAGGCTGTTCGGCAGATGCCTGTGATTCTGGAGATTC
SNX27-42-B_Fwd	AAGTTCTGTTTCAGGGCCCGCGGGTCGTGCGCATCGTCAAG
SNX27-135-B_Rev	ATGGTCTAGAAAGCTTTAttaTACAGATAACACTGTCAAGATCAATTCCTTCTCG
SNX27_pHAGE_Fwd	TTCGATgTCGAcGCGGACGAGGACGGGGAAG
SNX27_pHAGE_Rev	TTCGATggatccCTAATATTCCTCTTTTCTCCAC
SLC1A4_pHAGE_Fwd	TTCGATTCTAGAGAGAAGAGCAACGAGACC
SLC1A4_pHAGE_Rev	TTCGATgTCGAcTCACAGAACCGACTCCTTGG
OTULIN_C129A_Rev	CGTGGCCCTCAGTGCGGCGTAATTATCACCACGGAC
OTULIN_Y56F_Rev	TGCAGCACGGAACATGTCCTCCTC
OTULIN_ΔETSL_pEF_Rev	CTAGCGGCCGCTTACTCACACACTCTGAC
OTULIN_ΔETSL_pHAGE_Rev	CGATGTCGACTTACTCACACACTCTGACGG
OTULIN_348-B_Rev	ATGGTCTAGAAAGCTTTAttaCTCACACACTCTGACGGGGATGTTATAG
OTULIN_T350D_352-B_Rev	ATGGTCTAGAAAGCTTTAttaTAGACTGtcCTCCTCACACACTCTGACGGGGATG
OTULIN_E85R_Fwd	GCGTAGCTCCTagAATGGATATCATGGACTACTGC
OTULIN_E85R_Rev	CATGATATCCATTctAGGAGCTACGCTTAATCTCGG
OTULIN_D87R_Fwd	GCTCCTGAAATGcgtATCatggacTACTGCAAAAAAG
OTULIN_D87R_Rev	GCAGTAgtccatGATacgCATTTCAGGAGCTACGCTTAATC
OTULIN_E85R D87R_Rev	GCAGTAGTCCATGATTCTCATTCTAGGAGCTACGC
OTULIN_80-E85R-D87R_B_Fwd	AAGTTCTGTTTCAGGGCCCGTTAAGCGTAGCTCCTagAATGcgtATC
OTULIN_D90R_fwd	GAAATGgatATCatgCGcTACTGCAAAAAAGAATG
OTULIN_D90R_Rev	CTTTTTTGCAGTAgCGcatGATatcCATTTCAGGAG
OTULIN_E209R_Fwd	GAGAACTGCTAGaGCAAGACAGATAGCTTGTGATG
OTULIN_E209R_Rev	CTATCTGTCTTGCtCTAGCAGTTCTCATTTCAGCC
SNX27_R58E_Fwd	CGGCTTCAACGTGGAgGGCCAAGTGAGCGAGggcg
SNX27_R58E_Rev	GCTCACTTGGCCcTCCACGTTGAAGCCGTAGCCGGACTC
SNX27_G64E_Fwd	CAAGTGAGCGAGgaggggcaaCTGCGGAGCATCAACG
SNX27_G64E_Rev	GCTCCGCAGttgcccctcCTCGCTCACTTGGCC
SNX27_G65A_Fwd	GTGAGCGAGggcgCgcaaCTGCGGAGCATCAACGGGG
SNX27_G65A_Rev	GATGCTCCGCAGttgcGcgccCTCGCTCACTTGGCCccg
SNX27_R100E_Fwd	CAAGGGGGACGAGATCCTGGAGGTGAACCACGTG
SNX27_R100E_Rev	CTCCAGGATCTCGTCCCCCTTGCGCACCCC
rhoGEF_41-S_Fwd	GCGAACAGATCGGTGGTACCGGTCTGGTTCAGCGTTG
rhoGEF_123-B_Rev	ATGGTCTAGAAAGCTTTAttaGCTGCTACCCAGCAGGGTC
synten1_108-S_Fwd	GCGAACAGATCGGTGGTGCCGAAATCAAACAGGGTATTCG
synten1_196-B_Rev	ATGGTCTAGAAAGCTTTAttaCGGACGATCACGAATGGTCATG
NHERF1_150-S_Fwd	GCGAACAGATCGGTGGTCTGCGTCCGCGTCTGTGTA
NHERF1_235-B_Rev	ATGGTCTAGAAAGCTTTAttaGGTTTCACGATCAACAACCAGCA
LNX2_465-S_Fwd	GCGAACAGATCGGTGGTCAAGAGAAACACATCACCGTGAAAAAA
LNX2-560-B_Rev	ATGGTCTAGAAAGCTTTAttaTTCTTCAACAATCTGAACTTCCAGTGC
PSD95_155-S_Fwd	GCGAACAGATCGGTGGTGCCGAAAAAGTGATGGAAATCAAACTG
PSD95_249-B_Rev	ATGGTCTAGAAAGCTTTAttaTGCATTGCTCGGTTTGGCAACTT
PSD95_309-S_Fwd	GCGAACAGATCGGTGGTGAATTTCGTGAACCGCGTCGTAT
PSD95_413-B_Rev	ATGGTCTAGAAAGCTTTAttaGTTCATCAGCTGTTCGCGCAG
MUPP1_1346-S_Fwd	GCGAACAGATCGGTGGTGGTGAACTGCATATGATCGAACTG
MUPP1_1436-B_Rev	ATGGTCTAGAAAGCTTTAttaCACGGCATCTTTATTGCGAATGAAG
SHANK1_654-S_Fwd	GCGAACAGATCGGTGGTGGGAGCGATTACATCATTAAGGAGAAGAC
SHANK1_763-B_Rev	ATGGTCTAGAAAGCTTTAttaATCCATGTCCGGGTGCCTGGTG
SHANK2_293-S_Fwd	GCGAACAGATCGGTGGTAGTGACTGCATTATTGAGGAGAAGACGGT
SHANK2_347-B_Rev	ATGGTCTAGAAAGCTTTAttaGTCGGGGTCCAGATTCCTGGTC
SHANK3_563-S_Fwd	GCGAACAGATCGGTGGTAGCGATTATGTCATTGATGACAAAGTGGCTG
SHANK3_671-B_Rev	ATGGTCTAGAAAGCTTTAttaGTCCTCTTCTGGCTTCCTTGTCACAG

Supplementary Table 2: Oligonucleotide primer sequences for cloning and mutagenesis

RhoGEF_PDZ_41-123	ACCGGTCTGGTTCAGCGTTGTGTTATTATTCAGAAAGATCAGCATGGCTTTGGTTTTACCGTTAGCGGTGAT<br>CGTATTGTTCTGGTGCAGAGCGTTCGTCCTGGTGGTGCAGCAATGAAAGCCGGTGTTAAAGAAGGTGATCGC<br>ATTATCAAAGTTAACGGCACCATGGTTACCAATAGCAGCCATCTGGAAGTTGTGAAACTGATTAAAAGCGGT<br>GCATATGTTGCACTGACCCTGCTGGGTAGCAGC
PSD95_PDZ2	GCCGAAAAAGTGATGGAAATCAAACTGATCAAAGGTCCGAAAGGTCTGGGTTTTAGCATTGCCGGTGGTGTT<br>GGTAATCAGCATATTCCGGGTGATAATAGCATTTACGTGACCAAAATTATCGAAGGTGGTGCAGCACATAAA<br>GATGGTCGTCTGCAGATTGGTGATAAAATTCTGGCAGTTAATAGCGTTGGTCTGGAAGATGTTATGCATGAA<br>GATGCAGTTGCAGCACTGAAAAATACCTATGATGTGGTGTATCTGAAAGTTGCCAAACCGAGCAATGCA
PSD95_PDZ3	GAATTTCGTGAACCGCGTCGTATTGTTATTCATCGTGGTAGCACCGGTCTGGGTTTTAACATTGTTGGTGGT<br>GAAGATGGTGAAGGCATCTTTATTAGCTTTATTCTGGCAGGCGGTCCGGCAGATCTGAGCGGTGAACTGCGT<br>AAAGGTGATCAGATTCTGAGCGTTAATGGTGTTGATCTGCGTAATGCAAGCCATGAACAGGCAGCAATTGCA<br>CTGAAAAATGCAGGTCAGACCGTTACCATTATTGCACAGTATAAACCGGAAGAATATAGCCGCTTTGAAGCC<br>AAAATTCATGATCTGCGCGAACAGCTGATGAAC
LNX_PDZ3	CAAGAGAAACACATCACCGTGAAAAAAGAACCGCATGAAAGCCTGGGTATGACCGTTGCCGGTGGTCGTGGT<br>AGCAAAAGCGGTGAACTGCCGATTTTTGTTACCAGCGTTCCGCCTCATGGTTGTCTGGCACGTGATGGTCGT<br>ATTAAACGTGGTGATGTTCTGCTGAATATTAACGGTATTGATCTGACCAATCTGAGCCATAGCGAAGCAGTT<br>GCAATGCTGAAAGCAAGCGCAGCAAGTCCGGCAGTTGCACTGAAAGCACTGGAAGTTCAGATTGTTGAAGAA
NHERF1_PDZ2	CTGCGTCCGCGTCTGTGTACCATGAAAAAAGGTCCGAGCGGTTATGGTTTTAATCTGCATAGCGATAAAAGC<br>AAACCGGGTCAGTTTATTCGTAGCGTTGATCCGGATAGTCCTGCCGAAGCAAGCGGTCTGCGTGCACAGGAT<br>CGTATTGTTGAAGTTAATGGTGTTTGCATGGAAGGCAAACAGCATGGTGATGTTGTTAGCGCAATTCGTGCC<br>GGTGGTGATGAAACCAAACTGCTGGTTGTTGATCGTGAAACC
Syntenin1_PDZ1	GCCGAAATCAAACAGGGTATTCGTGAAGTTATTCTGTGCAAAGATCAGGATGGTAAAATTGGTCTGCGTCTG<br>AAAAGCGTGGATAATGGTATTTTTGTTCAGCTGGTTCAGGCAAATAGTCCGGCAAGCCTGGTGGGTCTGCGT<br>TTTGGTGATCAGGTTCTGCAGATTAATGGTGAAAATTGTGCAGGTTGGAGCAGCGATAAAGCACATAAAGTT<br>CTGAAACAGGCCTTTGGCGAAAAAATCACCATGACCATTCGTGATCGTCCG
MUPP1_PDZ8	GGTGAACTGCATATGATCGAACTGGAAAAAGGTCATAGCGGTCTGGGTCTGAGCCTGGCAGGTAATAAAGAT<br>CGTAGCCGTATGAGCGTTTTTATTGTTGGTATTGATCCGAATGGTGCAGCAGGTAAAGATGGTCGTCTGCAG<br>ATTGCAGATGAACTGCTGGAAATTAATGGCCAGATTCTGTATGGTCGTAGCCATCAGAATGCAAGCAGCATT<br>ATCAAATGTGCACCGAGCAAAGTGAAAATCATCTTCATTCGCAATAAAGATGCCGTG

Supplementary Table 3: Codon-optimised sequences for PDZ domains

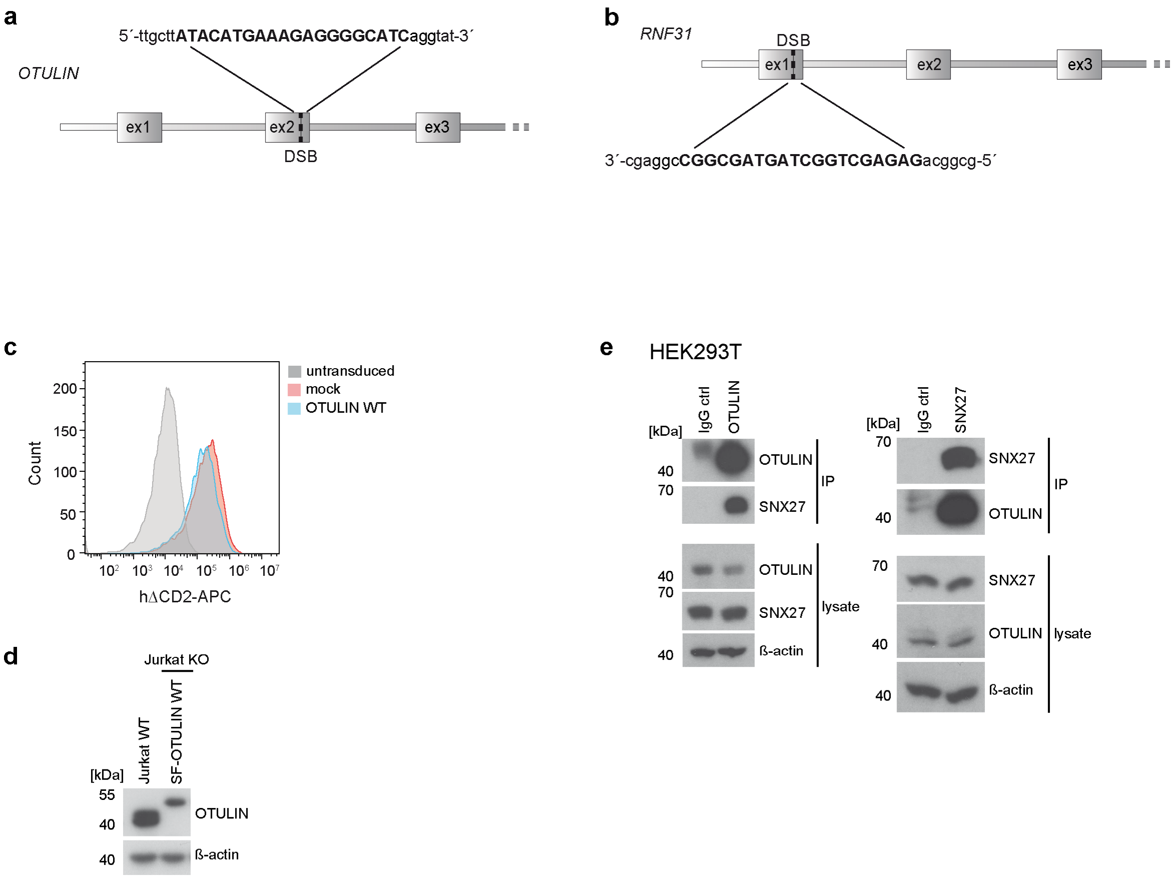

Supplementary Figure 1: (**a, b**) Schematic representations of the *OTULIN* and *RNF31* (HOIP) genes. Target sequences of the sgRNAs in exon 2 (OTULIN) and exon1 (RNF31) are depicted in bold type. (**c**) The transduction efficiency of mock and SF-OTULIN WT constructs into OTULIN KO Jurkat T cells was determined by co-expression of the cell surface marker ΔCD2 using flow cytometry. (**d**) Expression of SF-OTULIN in reconstituted OTULIN KO cells was assessed by WB and compared to endogenous OTULIN expression in parental Jurkat T cells. (**e**) OTULIN-IP and SNX27-IP from extracts of HEK293T cells. Interaction of SNX27 and OTULIN was analyzed by WB. Source data are provided as a Source Data file.

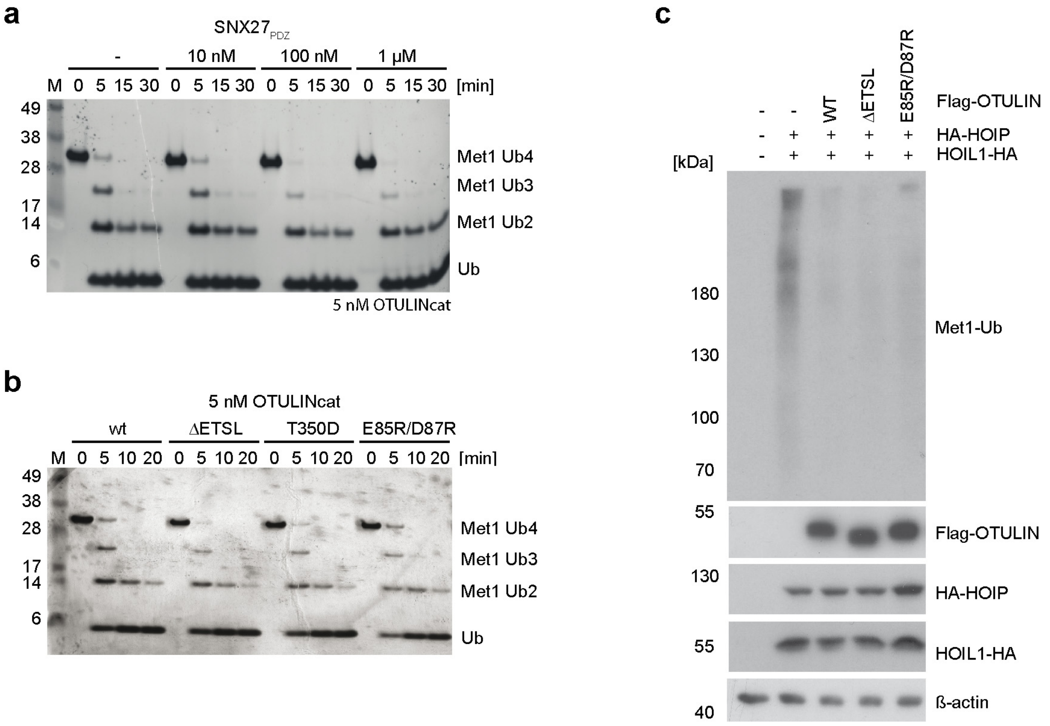

Supplementary Figure 2: (**a**) 5 nM OTULINcat (aa 80-352) was incubated with Met1-linked tetraUb in the presence of increasing $SNX27_{PDZ}$ amounts (aa 40-135). TetraUb chain cleavage was visualized on Coomassie-stained SDS-PAGE gels. (**b**) 5 nM OTULINcat (aa 80-352) WT or SNX27-binding mutants were incubated with Met1-linked tetraUb. TetraUb chain cleavage was visualized on Coomassie-stained SDS-PAGE gels. (**c**) HEK293 cells were transfected with HA-HOIP and HOIL1-HA alone or together with Flag-OTULIN WT or SNX27-binding mutants. Effects of OTULIN expression on LUBAC-catalyzed Met1-Ub chain accumulation was monitored by WB. Source data are provided as a Source Data file.

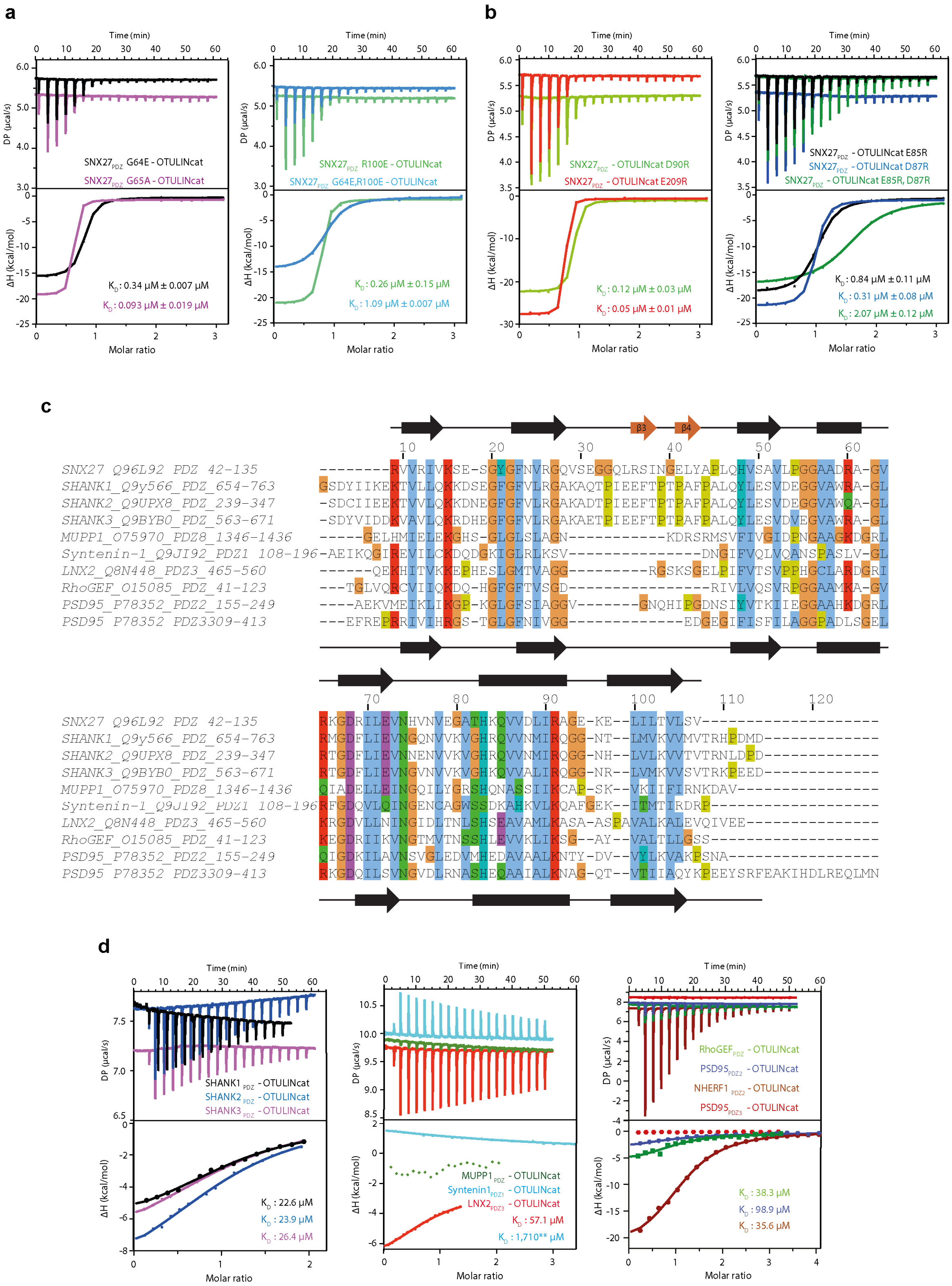

Supplementary Figure 3: (**a**) ITC data for structure-guided mutations within SNX27 at the second interface that reduce the affinity to OTULIN. (**b**) ITC data as in (a) for mutations in OTULIN that disrupt the second interface with SNX27. (**c**) Structure-based sequence alignment for all PDZ domains used in this study with the corresponding domain boundaries and UniProt accession numbers shown for each

PDZ domain. Secondary structure elements for the SNX27 PDZ domain are shown on top, with the corresponding β3-β4 insertion colored in orange. Other PDZ domains either lack this insertion or in the case of SHANKs 1-3 contain an unstructured loop. Secondary structure for SHANK1 PDZ (PDB ID: 3L4F) are shown on the bottom of the alignment. (**d**) ITC data for various closely related PDZ domains titrated against OTULIN.

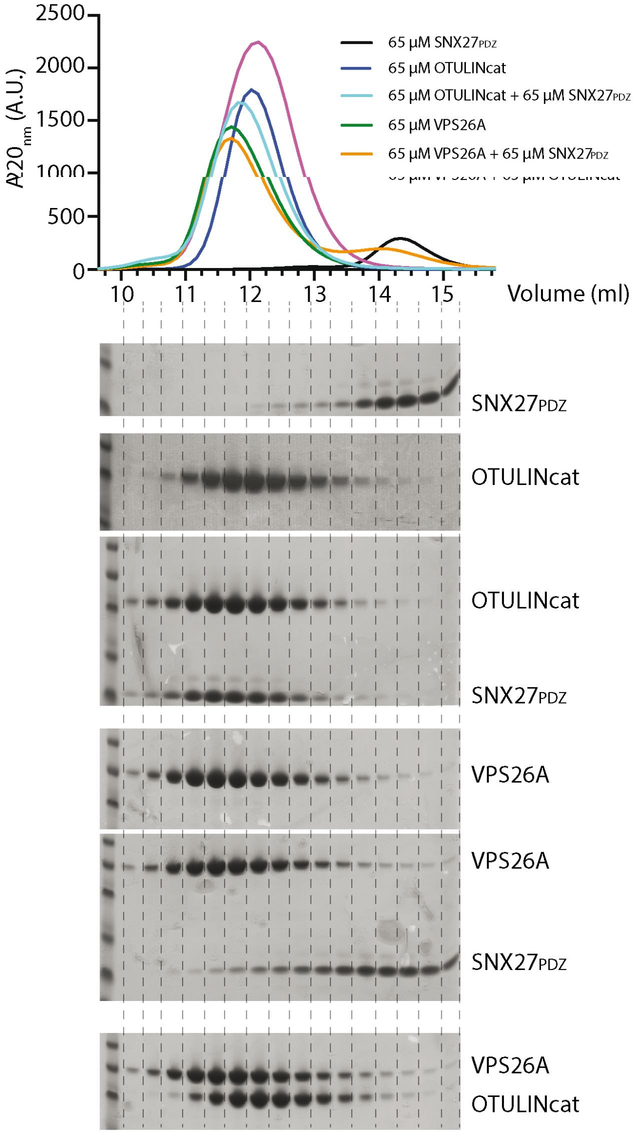

Supplementary Figure 4: Analytical size exclusion chromatography profile of $SNX27_{PDZ}$ (black), OTULINcat (blue), VPS26A (green) and corresponding equimolar complexes of OTULINcat + $SNX27_{PDZ}$ (cyan), VPS26A + OTULINcat (magenta) and VPS26A + $SNX27_{PDZ}$ (orange). Coomassie-stained SDS-PAGE gels below show protein-containing fractions.

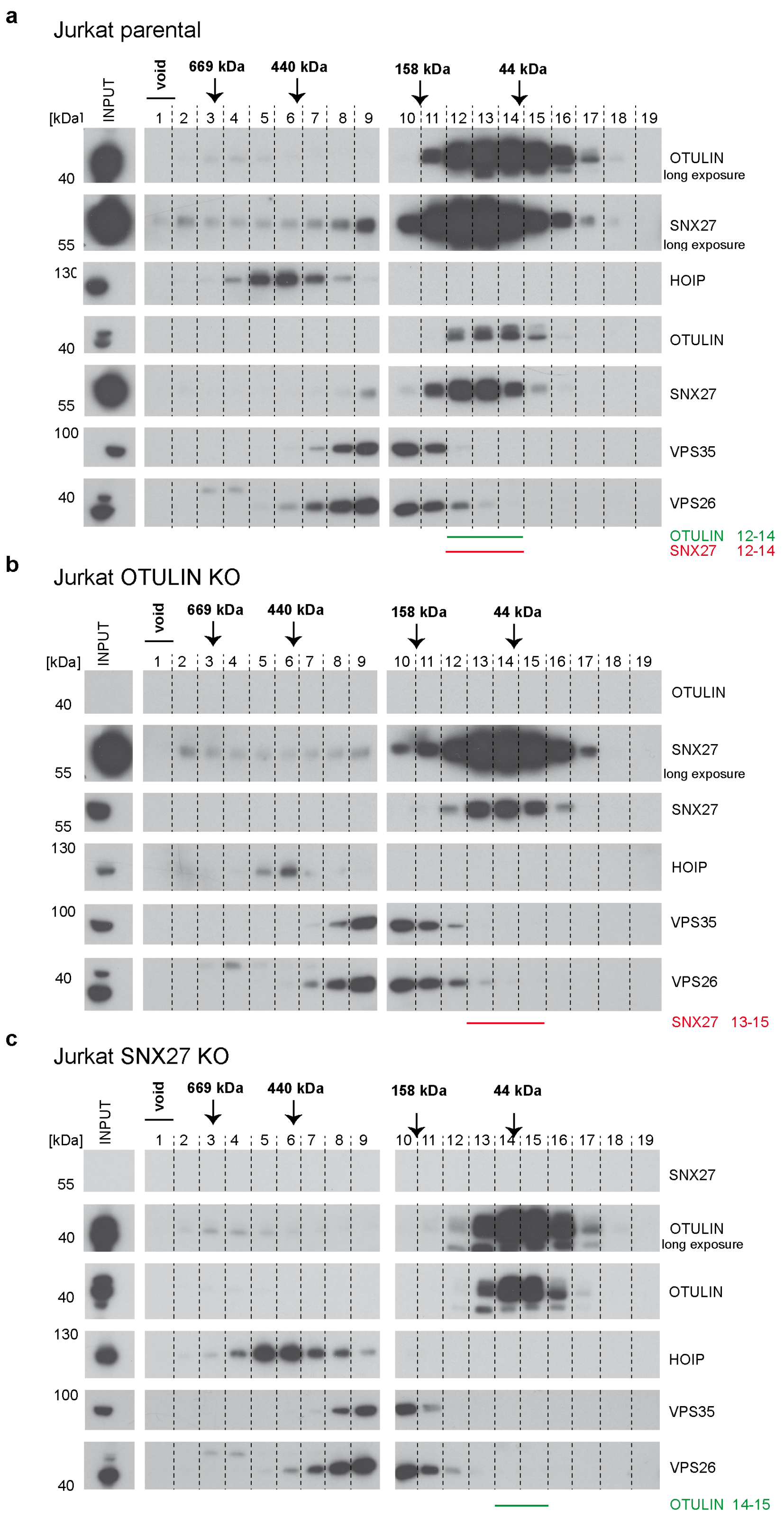

Supplementary Figure 5: (**a**) Extracts from Jurkat T cells were fractionated by size exclusion chromatography using a Superdex 200 column. Elution profiles of endogenous proteins (OTULIN, SNX27, HOIP, VPS35 and VPS26) were determined by WB. Peak elution of molecular weight standards is depicted at the top. (**b**) Extracts from OTULIN KO Jurkat T cells were fractionated by size exclusion

chromatography as in a. (**c**) Extracts from SNX27 KO Jurkat T cells were fractionated by size exclusion chromatography as in a. Source data are provided as a Source Data file.

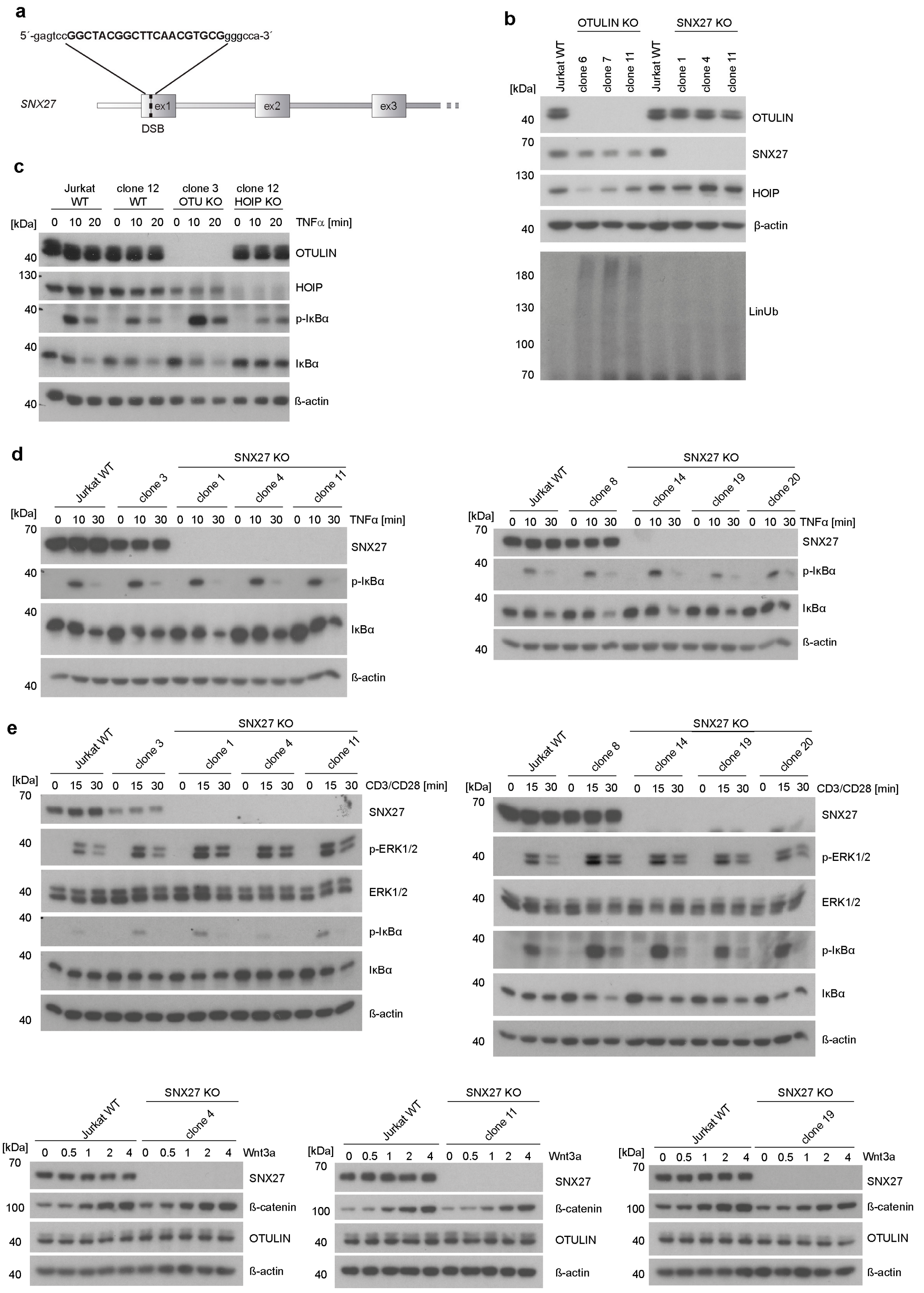

Supplementary Figure 6: (**a**) Schematic representation of the *SNX27* gene. Target sequence of the sgRNA in exon 1 is depicted in bold type. (**b**) SNX27 deficiency does not affect accumulation of Met1-linked ubiquitin chains. Extracts of WT, OTULIN-deficient or SNX27-deficient Jurkat T cells were analyzed by WB for the accumulation of Met1-linked ubiquitin chains and for the expression of HOIP,

OTULIN and SNX27. (**c**) WT (parental, clone 12), OTULIN KO and HOIP KO Jurkat T cells were stimulated with TNFα (20 ng/ml) for the indicated times. Extracts were analyzed for NF-κB signaling by WB using antibodies against p-IκBα and IκBα. (**d**) SNX27-deficient Jurkat T cells were stimulated with TNFα (20 ng/ml) for the indicated times and analyzed for NF-κB signaling by WB. (**e**) SNX27-deficient Jurkat T cells were treated with anti-CD3 and anti-CD28 for the indicated times. NF-κB and MAPK signaling was monitored by WB. (**f**) SNX27-deficient Jurkat T cells were treated with recombinant Wnt3a for the indicated times. Lysates were analyzed for β-catenin stabilization by WB. Source data are provided as a Source Data file.

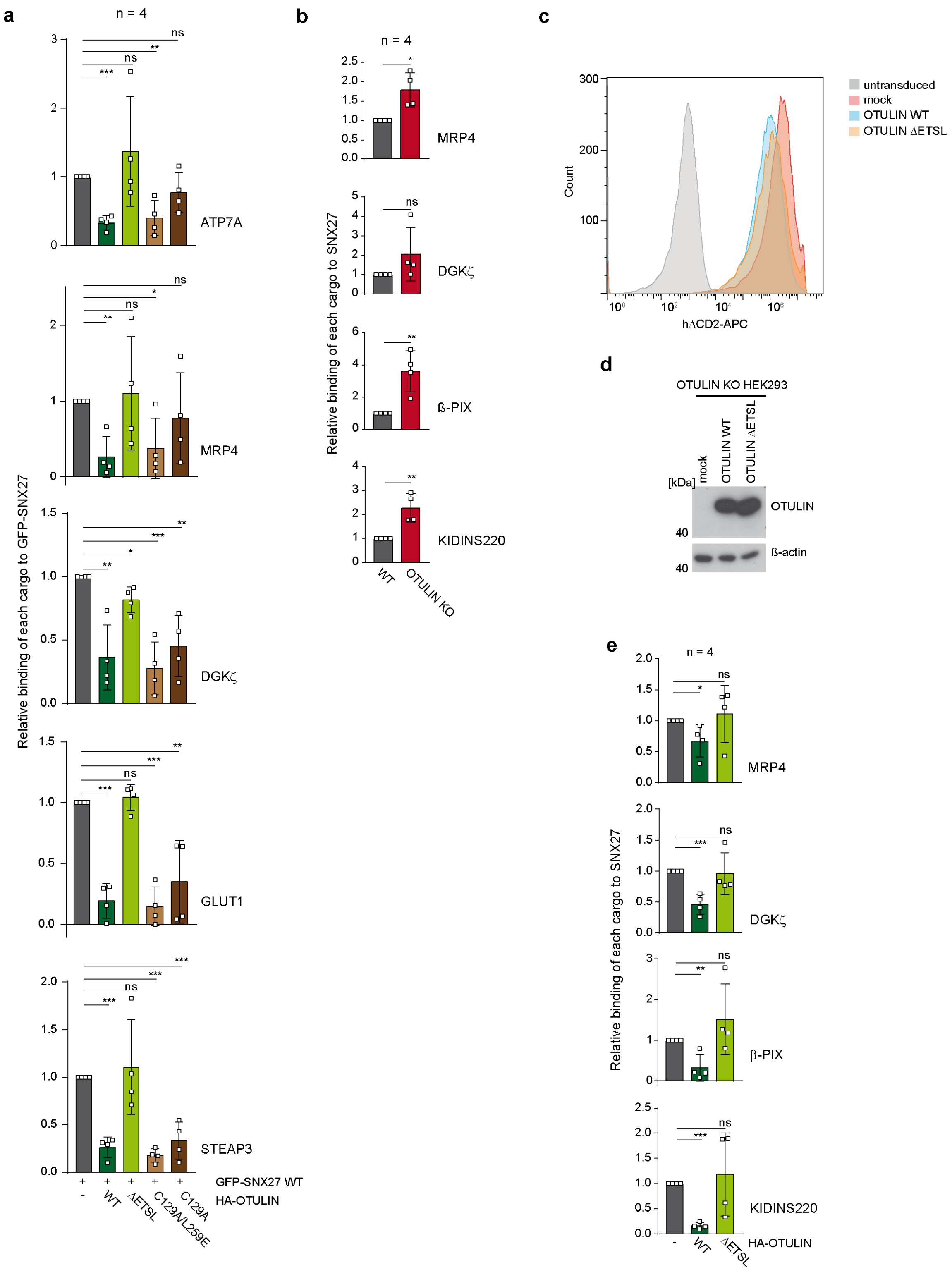

Supplementary Figure 7: (**a**) Binding of each cargo to GFP-SNX27 (see Figure 7d) was quantified in four independent experiments. Bound cargos after GFP-Trap were normalized to GFP-SNX27 after PD. Graphs depict the mean ± SD. Two-tailed p-values: ns not significant, *p ≤ 0.05, ** p ≤ 0.01, ***p ≤ 0.001 by unpaired t-test. (**b**) Binding of each cargo to SNX27 (see Figure 7e) was quantified in four independent experiments. Bound cargos after SNX27-IP were normalized to SNX27 after IP. Graphs

depict the mean ± SD. Two-tailed p-values: ns not significant, *p ≤ 0.05, **p ≤ 0.01 by unpaired t-test. (**c**) Lentiviral transduction efficiency after infection of mock and OTULIN constructs into OTULIN KO HEK293 cells was determined by the co-expressed cell surface marker ΔCD2 using flow cytometry. (**d**) Expression of OTULIN WT and OTULIN ΔETSL in reconstituted OTULIN KO cells was assessed by WB (**e**) Binding of each cargo to SNX27 (see Figure 7f) was quantified in four independent experiments. Bound cargos after SNX27-IP were normalized to SNX27 after IP. Graphs depict the mean ± SD. Two-tailed p-values: ns not significant, *p ≤ 0.05, **p ≤ 0.01, ***p ≤ 0.001 by unpaired t-test. Source data are provided as a Source Data file.

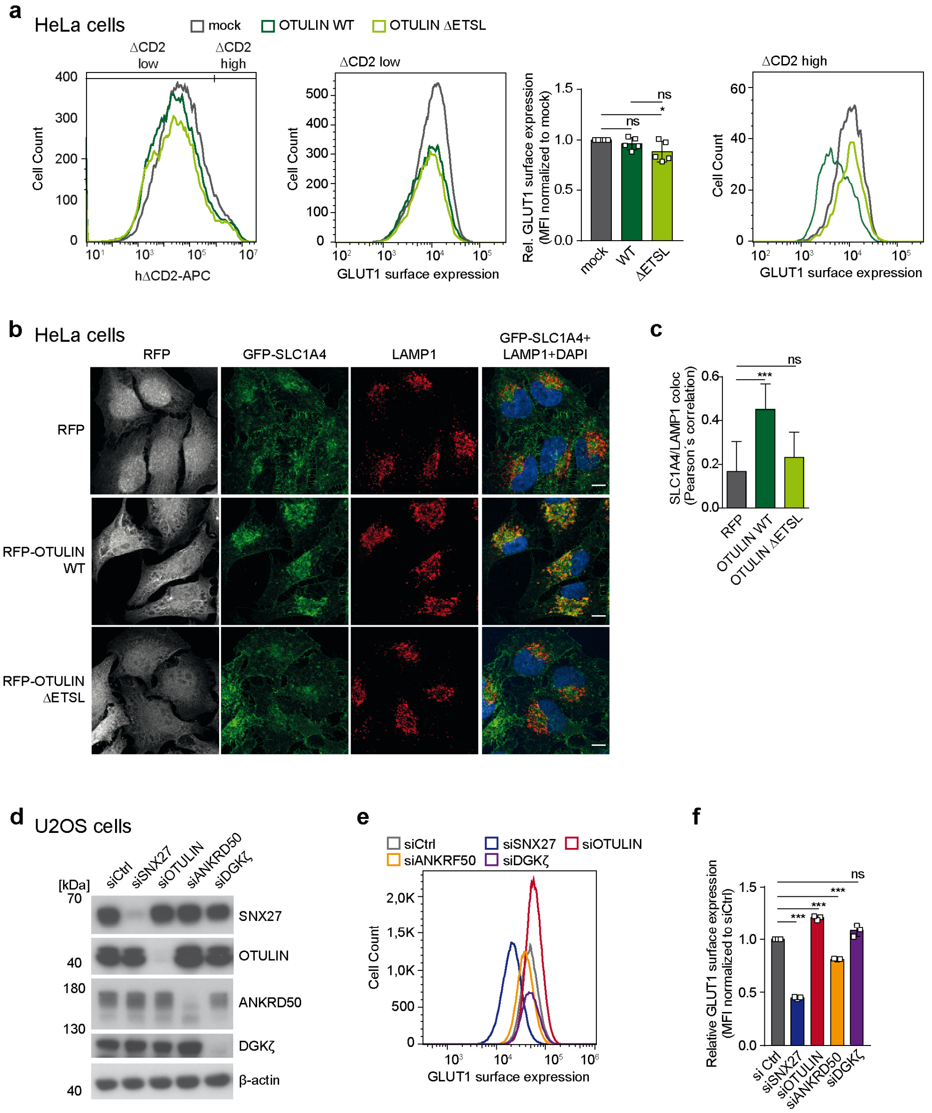

Supplementary Figure 8: (**a**) GLUT1 surface staining in mock, OTULIN WT and OTULIN ΔETSL transfected HeLa cells was performed using GLUT1.RBD.GFP reagent on living cells. GLUT1 surface expression was analyzed in untransfected (ΔCD2-low) and in transfected (ΔCD2-high) cells by FACS (left). Relative GLUT1 surface expression was determined as depicted in the histogram and changes in median fluorescence intensity (MFI) were normalized to mock. Graphs represent the mean ± SD of five independent experiments. Two-tailed p-values: ns not significant, *p ≤ 0.05, ***p ≤ 0.001 by unpaired t-test. (**b**) HeLa cells were lentivirally transduced with GFP-SLC1A4 (green) and RFP, RFP-OTULIN or RFP-OTULIN ΔETSL (grey) and stained for endogenous LAMP1 (red). Co-localization of SLC1A4/LAMP1 was analyzed by confocal microscopy. Scale bars: 10µm. (**c**) Co-localization was quantified by determination of Pearson's correlation analyzing at least 14 random pictures and imaging of more than 100 cells for each condition. Graphs depict the mean ± SD. Two-tailed p-values: ns not significant, ***p ≤ 0.001 by unpaired t-test. (**d**) Knock-down in U2OS cells after transfection of siSNX27, siOTULIN, siANKRD50 or siDGKζ was verified by WB. (**e**) GLUT1 surface staining in U2OS cells after transfection of siSNX27, siOTULIN, siANKRD50 or siDGKζ was performed using GLUT1.RBD.GFP reagent on living cells by flow cytometry. (**f**) Changes in median fluorescence intensity (MFI) were normalized to control siRNA to calculate relative GLUT1 surface expression. Graphs represent the mean ± SD of three independent experiments. Two-tailed p-values: ns not significant, ***p ≤ 0.001 by unpaired t-test. Source data are provided as a Source Data file.
