# Supplementary material for: Regulation of the endosomal SNX27-retromer by OTULIN
Source: Nat Commun. 2019 Sep 20;10:4320. doi: 10.1038/s41467-019-12309-z (PMC6754446; doi:10.1038/s41467-019-12309-z)
Supplement: Supplementary file 3 — Reporting Summary [file 41467_2019_12309_MOESM3_ESM.pdf]

nature research

Corresponding author(s): DBPR NCOMMS-19-01451-T

Last updated by author(s): Jul 18, 2019

Reporting Summary

Nature Research wishes to improve the reproducibility of the work that we publish. This form provides structure for consistency and transparency in reporting. For further information on Nature Research policies, see Authors & Referees and the Editorial Policy Checklist.

Statistics

For all statistical analyses, confirm that the following items are present in the figure legend, table legend, main text, or Methods section.

n/a	Confirmed	
☐	☒	The exact sample size (*n*) for each experimental group/condition, given as a discrete number and unit of measurement
☐	☒	A statement on whether measurements were taken from distinct samples or whether the same sample was measured repeatedly
☐	☒	The statistical test(s) used AND whether they are one- or two-sided<br>*Only common tests should be described solely by name; describe more complex techniques in the Methods section.*
☒	☐	A description of all covariates tested
☐	☒	A description of any assumptions or corrections, such as tests of normality and adjustment for multiple comparisons
☐	☒	A full description of the statistical parameters including central tendency (e.g. means) or other basic estimates (e.g. regression coefficient) AND variation (e.g. standard deviation) or associated estimates of uncertainty (e.g. confidence intervals)
☐	☒	For null hypothesis testing, the test statistic (e.g. *F*, *t*, *r*) with confidence intervals, effect sizes, degrees of freedom and *P* value noted<br>*Give P values as exact values whenever suitable.*
☒	☐	For Bayesian analysis, information on the choice of priors and Markov chain Monte Carlo settings
☐	☒	For hierarchical and complex designs, identification of the appropriate level for tests and full reporting of outcomes
☐	☒	Estimates of effect sizes (e.g. Cohen's *d*, Pearson's *r*), indicating how they were calculated

Our web collection on statistics for biologists contains articles on many of the points above.

Software and code

Policy information about availability of computer code

Data collection	LC-MS/MS: Dionex Ultimate 3000 nano-ultra high pressure reverse phase chromatography coupled on-line to a Q Exactive High Field (HF) mass spectrometer (Thermo Scientific)<br>ITC: Auto iTC200 instrument (Malvern Instruments, Malvern, UK)<br>Crystallization data collection: Diamond Light source beamline IO3<br>Flow Cytometry: Attune Acoustic Focusing Cytometer; Attune NxT<br>Leica SP5 confocal laser scanning microscope (Leica Microsystems)
Data analysis	LC-MS/MS: UniProtKB human sequence data base (retrieved 15.10.2014), MaxQuant Software (v1.5.2), Perseus (v.1.5.5.3)<br>ITC: PEAQ Analysis software (Malvern Instruments, Malvern, UK)<br>Crystallization data collection and refinement: Diffraction images were processed using xia2 4 and manually scaled using AIMLESS. The structure of OTULINcat C129A bound to SNX27PDZ was determined by molecular replacement using PHASER placing SNX27PDZ (PDB ID: 4P2A) and OTULINcat (PDB ID: 3ZNZ). Iterative rounds of model building and refinement were performed with COOT and PHENIX, respectively. Data collection and refinement statistics can be found in Table 1. All structure figures were generated with Pymol (www.pymol.org).<br>Flow Cytometry: FlowJo 10.4.2<br>IF: Fiji/ImageJ software<br>Data visualization: GraphPad Prism 7; Adobe Photoshop CS6; Adobe Illustrator CS6

For manuscripts utilizing custom algorithms or software that are central to the research but not yet described in published literature, software must be made available to editors/reviewers. We strongly encourage code deposition in a community repository (e.g. GitHub). See the Nature Research guidelines for submitting code & software for further information.

Data

Policy information about availability of data

All manuscripts must include a data availability statement. This statement should provide the following information, where applicable:

- Accession codes, unique identifiers, or web links for publicly available datasets
- A list of figures that have associated raw data
- A description of any restrictions on data availability

The mass spectrometry proteomics data have been deposited to the ProteomeXchange Consortium via the PRIDE partner repository with the dataset identifier: PXD012082. Coordinates for the OTULIN–SNX27 structure have been deposited in the PDB with accession number 6SAK. Source data underlying Figs. 6–8 and Supplementary Figs. 7 and 8 are provided as a Source Data file with the paper. All other data are available from the authors upon reasonable requests.

Field-specific reporting

Please select the one below that is the best fit for your research. If you are not sure, read the appropriate sections before making your selection.

[X] Life sciences [ ] Behavioural & social sciences [ ] Ecological, evolutionary & environmental sciences

For a reference copy of the document with all sections, see nature.com/documents/nr-reporting-summary-flat.pdf

Life sciences study design

All studies must disclose on these points even when the disclosure is negative.

Sample size	No statistical analysis methods were used to predetermine sample size estimates.<br>LC-MS/MS analysis was performed in biological quadruplicate.<br>Determination of GLUT1 cell surface expression: At least three independent experiments.
Data exclusions	No data were excluded from the analyses.
Replication	All experimental findings were reliably reproduced as indicated in the figure legends.
Randomization	No randomization was performed.
Blinding	Blinding was not performed.

Behavioural & social sciences study design

All studies must disclose on these points even when the disclosure is negative.

Study description	*Briefly describe the study type including whether data are quantitative, qualitative, or mixed-methods (e.g. qualitative cross-sectional, quantitative experimental, mixed-methods case study).*
Research sample	*State the research sample (e.g. Harvard university undergraduates, villagers in rural India) and provide relevant demographic information (e.g. age, sex) and indicate whether the sample is representative. Provide a rationale for the study sample chosen. For studies involving existing datasets, please describe the dataset and source.*
Sampling strategy	*Describe the sampling procedure (e.g. random, snowball, stratified, convenience). Describe the statistical methods that were used to predetermine sample size OR if no sample-size calculation was performed, describe how sample sizes were chosen and provide a rationale for why these sample sizes are sufficient. For qualitative data, please indicate whether data saturation was considered, and what criteria were used to decide that no further sampling was needed.*
Data collection	*Provide details about the data collection procedure, including the instruments or devices used to record the data (e.g. pen and paper, computer, eye tracker, video or audio equipment) whether anyone was present besides the participant(s) and the researcher, and whether the researcher was blind to experimental condition and/or the study hypothesis during data collection.*
Timing	*Indicate the start and stop dates of data collection. If there is a gap between collection periods, state the dates for each sample cohort.*
Data exclusions	*If no data were excluded from the analyses, state so OR if data were excluded, provide the exact number of exclusions and the rationale behind them, indicating whether exclusion criteria were pre-established.*
Non-participation	*State how many participants dropped out/declined participation and the reason(s) given OR provide response rate OR state that no participants dropped out/declined participation.*
Randomization	*If participants were not allocated into experimental groups, state so OR describe how participants were allocated to groups, and if allocation was not random, describe how covariates were controlled.*

Ecological, evolutionary & environmental sciences study design

All studies must disclose on these points even when the disclosure is negative.

Study description	*Briefly describe the study. For quantitative data include treatment factors and interactions, design structure (e.g. factorial, nested, hierarchical), nature and number of experimental units and replicates.*
Research sample	*Describe the research sample (e.g. a group of tagged Passer domesticus, all Stenocereus thurberi within Organ Pipe Cactus National Monument), and provide a rationale for the sample choice. When relevant, describe the organism taxa, source, sex, age range and any manipulations. State what population the sample is meant to represent when applicable. For studies involving existing datasets, describe the data and its source.*
Sampling strategy	*Note the sampling procedure. Describe the statistical methods that were used to predetermine sample size OR if no sample-size calculation was performed, describe how sample sizes were chosen and provide a rationale for why these sample sizes are sufficient.*
Data collection	*Describe the data collection procedure, including who recorded the data and how.*
Timing and spatial scale	*Indicate the start and stop dates of data collection, noting the frequency and periodicity of sampling and providing a rationale for these choices. If there is a gap between collection periods, state the dates for each sample cohort. Specify the spatial scale from which the data are taken*
Data exclusions	*If no data were excluded from the analyses, state so OR if data were excluded, describe the exclusions and the rationale behind them, indicating whether exclusion criteria were pre-established.*
Reproducibility	*Describe the measures taken to verify the reproducibility of experimental findings. For each experiment, note whether any attempts to repeat the experiment failed OR state that all attempts to repeat the experiment were successful.*
Randomization	*Describe how samples/organisms/participants were allocated into groups. If allocation was not random, describe how covariates were controlled. If this is not relevant to your study, explain why.*
Blinding	*Describe the extent of blinding used during data acquisition and analysis. If blinding was not possible, describe why OR explain why blinding was not relevant to your study.*

Did the study involve field work? ☐ Yes ☐ No

Field work, collection and transport

Field conditions	*Describe the study conditions for field work, providing relevant parameters (e.g. temperature, rainfall).*
Location	*State the location of the sampling or experiment, providing relevant parameters (e.g. latitude and longitude, elevation, water depth).*
Access and import/export	*Describe the efforts you have made to access habitats and to collect and import/export your samples in a responsible manner and in compliance with local, national and international laws, noting any permits that were obtained (give the name of the issuing authority, the date of issue, and any identifying information).*
Disturbance	*Describe any disturbance caused by the study and how it was minimized.*

Reporting for specific materials, systems and methods

We require information from authors about some types of materials, experimental systems and methods used in many studies. Here, indicate whether each material, system or method listed is relevant to your study. If you are not sure if a list item applies to your research, read the appropriate section before selecting a response.

Materials & experimental systems

n/a	Involved in the study	
☐	☒	Antibodies
☐	☒	Eukaryotic cell lines
☒	☐	Palaeontology
☐	☒	Animals and other organisms
☒	☐	Human research participants
☒	☐	Clinical data

Methods

n/a	Involved in the study	
☒	☐	ChIP-seq
☐	☒	Flow cytometry
☒	☐	MRI-based neuroimaging

Antibodies

Antibodies used	anti-Actin (sc-1616 HRP), anti-ATP7A (sc-376467), anti-normal mouse IgG (sc-2025), anti-ERK (sc-514302, all Santa Cruz); anti-GFP (2555), anti-OTULIN (14127), anti-IκBα (4814), anti-phospho-IκBα (Ser32/36) (9246), anti-LAMP1 (15665), anti-phospho-ERK (Thr202/Tyr204) (9101), anti-normal rabbit IgG (2729), anti-EEA1 (3288, all CST); anti-SNX27 (ab77799), anti-VPS26 (ab23892), anti-VPS35 (ab157220), anti-MRP4 (ab15602), anti-DGKζ (ab105195), anti-GLUT1 (ab15309 and ab115730, all abcam); anti-Beta PIX (07-1450-I), anti-Met1-Ub (MABS199, all Millipore); anti-STEAP3 (17186-1-AP), anti-KIDINS220 (21856-1-AP, all Proteintech); anti-SNX27 (Gift from W. Hong); anti-Beta-catenin (610153, BD); anti-FLAG M2 (F3165, Sigma); anti-HOIP (MAB8039, R&D); anti-HA (Core facility monoclonal antibodies HMGU); anti-CD2-APC (17-0029-41, eBioscience).
Validation	All antibodies were validated for the application and species by their manufacturers.

Eukaryotic cell lines

Policy information about cell lines

Cell line source(s)	Jurkat T cells were obtained from the laboratory of L. Schmitz (University of Giessen) and verified by DSMZ. HeLa, HEK293 and HEK293T cells were obtained by DSMZ (RRID: CVCL_0030/CVCL_0045/CVCL_0063). U2OS cells (RRID: CVCL_0042) were purchased from ATCC (RRID: ATCC-HTB-96). MEF were generated from WT and OTULIN KO mice (Damgaard et al, 2016 Cell 166, 1215-1230 e1220)
Authentication	Jurkat T cells were authenticated by DSMZ.
Mycoplasma contamination	All cell lines were tested negative for mycoplasma contamination.
Commonly misidentified lines (See ICLAC register)	No commonly misidentified cell line was used.

Animals and other organisms

Policy information about studies involving animals; ARRIVE guidelines recommended for reporting animal research

Laboratory animals	C57BL/6J (Jackson, 000664)
Wild animals	The study did not involve wild animals.
Field-collected samples	The study did not involve samples collected from the field.
Ethics oversight	*Identify the organization(s) that approved or provided guidance on the study protocol, OR state that no ethical approval or guidance was required and explain why not.*

Note that full information on the approval of the study protocol must also be provided in the manuscript.

Flow Cytometry

Plots

Confirm that:

- [x] The axis labels state the marker and fluorochrome used (e.g. CD4-FITC).
- [x] The axis scales are clearly visible. Include numbers along axes only for bottom left plot of group (a 'group' is an analysis of identical markers).
- [ ] All plots are contour plots with outliers or pseudocolor plots.
- [x] A numerical value for number of cells or percentage (with statistics) is provided.

Methodology

Sample preparation	Determination of cell surface expression of Glucose transporter 1 (GLUT1)<br>100.000 cells were resuspended in 95 µl Buffer A (Complete culture medium, 0.1% NaN3 and 1 mM EDTA) and incubated with GLUT1.RBD.GFP (Metafora Biosystems) and with or without 0.25 µl anti-CD2-APC antibody (eBioscience, 17-0029-41) for 20 min at 37°C. After staining, cells were washed three times with Buffer B (PBS, 0.1% NaN3, 1 mM EDTA and 2% FCS) and analyzed for CD2 and GLUT1 surface expression by FACS.<br>For quantification of GLUT1 surface expression, median fluorescence intensities of the GLUT1.RBD.GFP signal were determined with the FlowJo software and normalized to mock. Data from at least three independent experiments (n=3) were analyzed and depicted as mean ± standard deviation (SD). Statistical analysis was performed using the unpaired Student´s t-test (GraphPad PRISM 7).
Instrument	Attune Acoustic Focusing Cytometer

Software	Attune Cytometric Software v2.1; FlowJo v10.4.2
Cell population abundance	not applicable
Gating strategy	Gate 1: FSC-A/SSC-A parameters were used for gating on living cells.<br>[Gate 2: CD2-APC parameter was used for gating on CD2-expressing cells.]<br>Gate 3: GLUT1.RBD.GFP parameter was used to determine GLUT1 expression on the cell surface.

☒ Tick this box to confirm that a figure exemplifying the gating strategy is provided in the Supplementary Information.
